# Pulmonary and Urologic Sarcoidosis as a Cause of Intermittent Fever of Unknown Origin

**DOI:** 10.7759/cureus.55709

**Published:** 2024-03-07

**Authors:** Diana Oliveira Miranda, José N Magalhães, Diogo Carvalho Sá, Patricia Neves, Fabienne Gonçalves

**Affiliations:** 1 Department of Medicine, Unidade Local de Saúde de Santo António, Porto, PRT; 2 Department of Pathology, Unidade Local de Saúde de Santo António, Porto, PRT; 3 Department of Semiology, Instituto de Ciências Biomédicas Abel Salazar, Universidade do Porto, Porto, PRT

**Keywords:** pulmonary sarcoidosis, sarcoidosis, fever of unknown origin, genitourinary sarcoidosis, urology sarcoidosis

## Abstract

Diagnosing fever of unknown origin (FUO) presents a substantial challenge due to its potential association with various diseases affecting different organs. In 1961, Petersdorf and Beeson initially defined FUO as a condition characterized by a temperature exceeding 38.3 °C on at least three occasions over a minimum three-week period. Despite a week of inpatient investigation, a definitive diagnosis remains unclear.

Sarcoidosis, a granulomatous disease impacting multiple systems, is among the causes of FUO. While the lungs are commonly affected, any organ can be involved, leading to diverse manifestations and clinical courses. Diagnosis relies on clinicopathologic findings and the exclusion of alternative causes of granulomatous disease. The hallmark of sarcoidosis is the development of granulomas in affected organs.

Here, we present the case of a 61-year-old man with a history of recurrent spontaneous periurethral abscesses who underwent multiple urological interventions. He developed FUO during hospitalization following treatment for the infectious condition.

## Introduction

There are about 200 described causes linked to the symptomatology of fever of unknown origin (FUO). The conventional classification of classical FUO causes, based on existing literature, divides them into four primary etiological categories: infections, neoplasms, non-infectious inflammatory diseases (NIID) like connective-tissue diseases and vasculitis, as well as miscellaneous conditions [1,2]. Additionally, some authors introduce undiagnosed or idiopathic FUO as a fifth category, accounting for approximately 10-50% of cases [3,4]. Some advocate that the latter represents the true essence of what is termed a fever of unknown origin. 

Sarcoidosis, a systemic disorder affecting various organs, retains its elusive etiology despite decades of extensive research. The exact origin of sarcoidosis remains uncertain, as the predominant theory revolves around a complex interplay of genetic predisposition and environmental factors as crucial contributors to its pathogenesis [5]. Recent epidemiological data indicates that African Americans face a three- to four-times higher risk of developing sarcoidosis compared to Caucasians, and is 10 times more prevalent in women than in men. Moreover, extrapulmonary manifestations of sarcoidosis affect up to 25-30% of patients. While the involvement of the genitourinary (GU) tract has traditionally been reported as rare (less than 1% of patients with sarcoidosis), the literature suggests that the incidence may, in fact, be considerably higher, with cases often going undetected or presenting symptoms that are not immediately attributable to the urinary tract [6].

The diagnosis of sarcoidosis is hinged upon the fulfillment of the following criteria: (1) the identification of noncaseating granuloma through histopathologic examination; (2) a clinically consistent presentation; and (3) the thorough exclusion of alternative causes of granulomatous inflammation [5-7]. Regarding clinical presentation, the symptoms can vary depending on the affected organs. Most sarcoidosis patients exhibit no symptoms [8]. GU symptoms may include renal colic, microscopic or macroscopic hematuria, and hydronephrosis. Obstructive uropathy can also result from sarcoid retroperitoneal lymphadenopathy or fibrosis [9]. Notably, the occurrence of fever is typically an uncommon manifestation in sarcoidosis, and its presence should prompt suspicion of a complication [4].

This report describes a case of fever of unknown origin (FUO) in a middle-aged man, showcasing an unusual and infrequent manifestation of sarcoidosis. Following the exclusion of other causes of granulomatous inflammation, the diagnosis of sarcoidosis involving both the pulmonary and genitourinary systems was established based on a combination of clinical and histological characteristics. This clinical report highlights a rare scenario where the genitourinary tract serves as the initial organ to display signs of disease involvement in sarcoidosis.

## Case presentation

**Figure 1:**
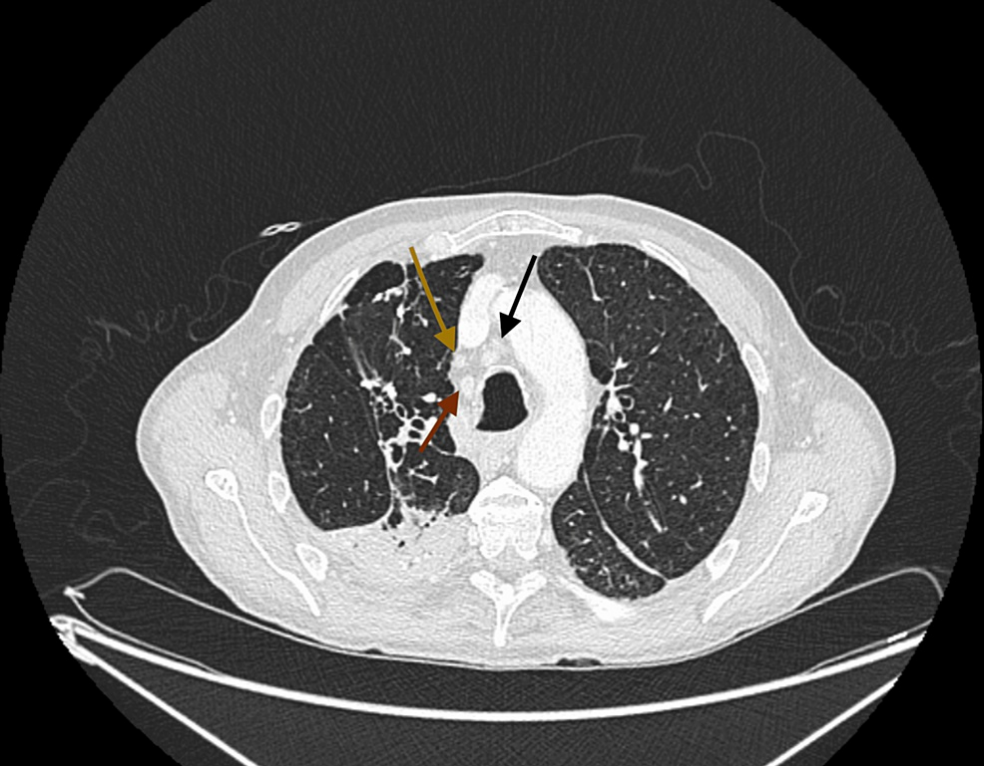
The CT thoracic tomography revealed multiple mediastinal lymphadenopathies red arrow: inferior pretracheal node on the right measuring 17 mm in short axis; yellow and black arrows: infracarinal node measuring 16 mm, and right hilar node measuring 11 mm

We introduce a 61-year-old man who has been followed at our center, specifically in the outpatient urology clinic, due to recurring urethral infections dating back to 2016. He had previously undergone a suprapubic cystostomy and penobulbar urethroplasty in 2016, followed by the drainage of a periurethral abscess connected to the urethra in 2019. In 2023, the patient was admitted to the urology inpatient department for the recurrence of a penile abscess, necessitating drainage and urethroplasty using oral mucosa. Post-surgery, the patient underwent a course of antibiotic therapy with cefuroxime and amikacin, leading to symptom improvement without isolation of any microorganisms. However, one week after completing the antibiotic course, he developed a fever, and the inflammatory parameters increased without a discernible new clinical focus. Initially presumed to be associated with a nosocomial infection, the patient underwent another round of antibiotics with imipenem and vancomycin. Despite this, the condition persisted, marked by sustained fever and elevated C-reactive protein (CRP) (>200 mg/dl) (Table 1). Several blood cultures were negative. Thoracoabdominopelvic computed tomography (CT) revealed the presence of multiple mediastinal and hilar lymph node formations exhibiting criteria indicative of adenomegaly (Figure 1). Fibrocicatricial changes were observed in both upper lobes, particularly prominent on the right side, displaying coarse calcifications and calcified micronodules, alongside evidence of traction bronchiectasis. These findings are suggestive of probable sequelae related to a previous granulomatous disease. 

**Table 1:** Results of the analytical study Results of the analytical study following two rounds of intravenous antibiotic therapy in the urology ward

Parameters	Values	Reference Range
White blood cells	10.65	4.8 – 10.8 (x 10^3/µL)
Neutrophils	6.95	2,00 - 7,50 (x 10^3/µL)
Eosinophils	0.12	0.00 – 0.49 (x 10^3/µL)
Basophils	0.03	0.0 – 0.1 (x 10^3/µL)
Lymphocytes	2.77	1.0 – 4.8 (x 10^3/µL)
Monocytes	0.72	0.12 – 0.80 (x 10^3/µL)
Platelets	398	150 – 350 (x 10^3/µL)
C-reactive protein	282.98	<3.0 (mg/dL)
Urea	44	15 – 39 (mg/dL)
Creatinine	0.77	0.70 – 1.30 (mg/dL)
Sodium	135	135 – 146 (mEq/L)
Potassium	4.9	3.5 – 5.1 (mEq/L)
Total bilirubin	0.35	0.3 – 1.2 (mg/dL)
Aspartate aminotransferase	13	12 – 40 (UI/L)
Alanine aminotransferase	12	7 – 40 (UI/L)
Gamma-glutamyl transferase	132	0 – 73 (UI/L)
Alkaline phosphatase	58	46 – 116 (UI/L)
Procalcitonin	0.01	0.000 - 0.050 (ng/mL)

Considering the lack of response to broad-spectrum antibiotics, persistent fever, elevated CRP levels, and the newly discovered findings on the CT scan, the patient was transferred to the internal medicine inpatient ward for a more comprehensive etiological investigation. 

In the internal medicine inpatient ward, upon reviewing the patient's medical history, he denied any previous episodes of anorexia, weight loss, fatigue, fever, hyperhidrosis, myalgias, skin lesions, changes in mucous membranes, eyes, or indications of pain or inflammation in the joints. A long-time smoker, he only complained of a persistently productive cough compatible with chronic bronchitis. He did not experience dyspnea or chest pain. The patient denied any history of contact with domestic animals or livestock, consumption of unpasteurized dairy products, or unpiped water. He had no recent travel history and reported no risky sexual behaviors. He had a history of tuberculosis in the past that was treated.

**Figure 2:**
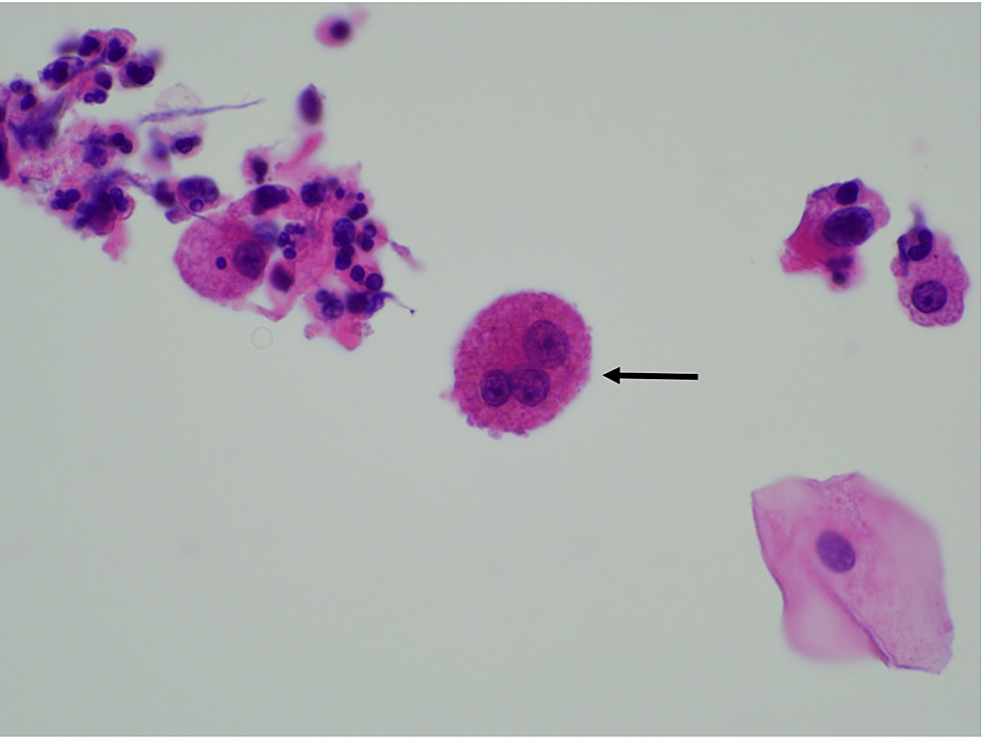
Cytological analysis of BAL and bronchial aspirate with identification of multinucleated giant cells H&E 600x magnification: multinucleated giant cells (arrow) BAL: bronchoalveolar lavage

**Figure 3:**
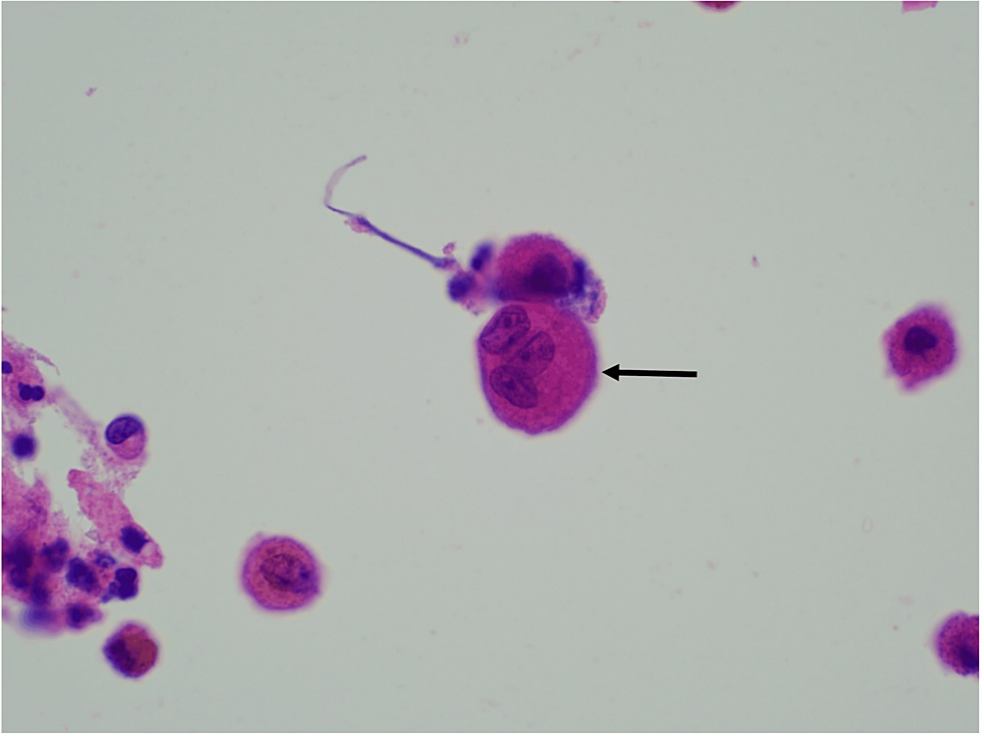
Cytological analysis of BAL and bronchial aspirate with identification of multinucleated giant cells H&E 600x magnification: multinucleated giant cells (arrow) BAL: bronchoalveolar lavage

**Figure 4:**
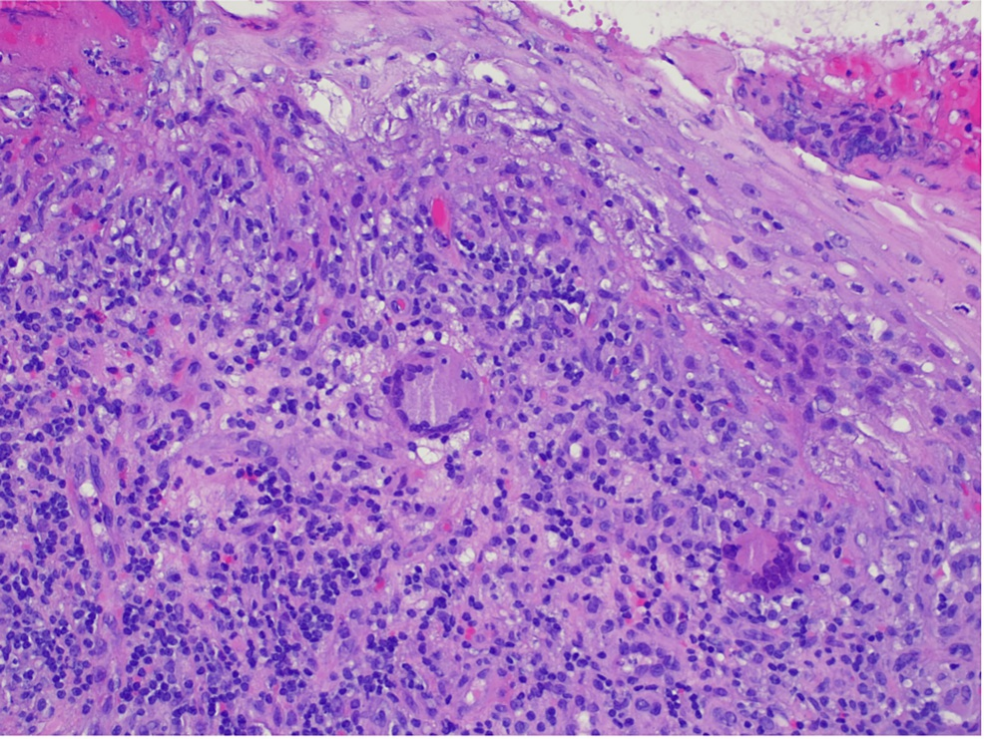
Anatomopathological evaluation of penobulbar urethra fragments H&E 200x magnification: lymphohistiocytic inflammatory cell infiltrate of the lamina propria of the urethral mucosa with scattered multinucleated giant cells (arrows)

**Figure 5:**
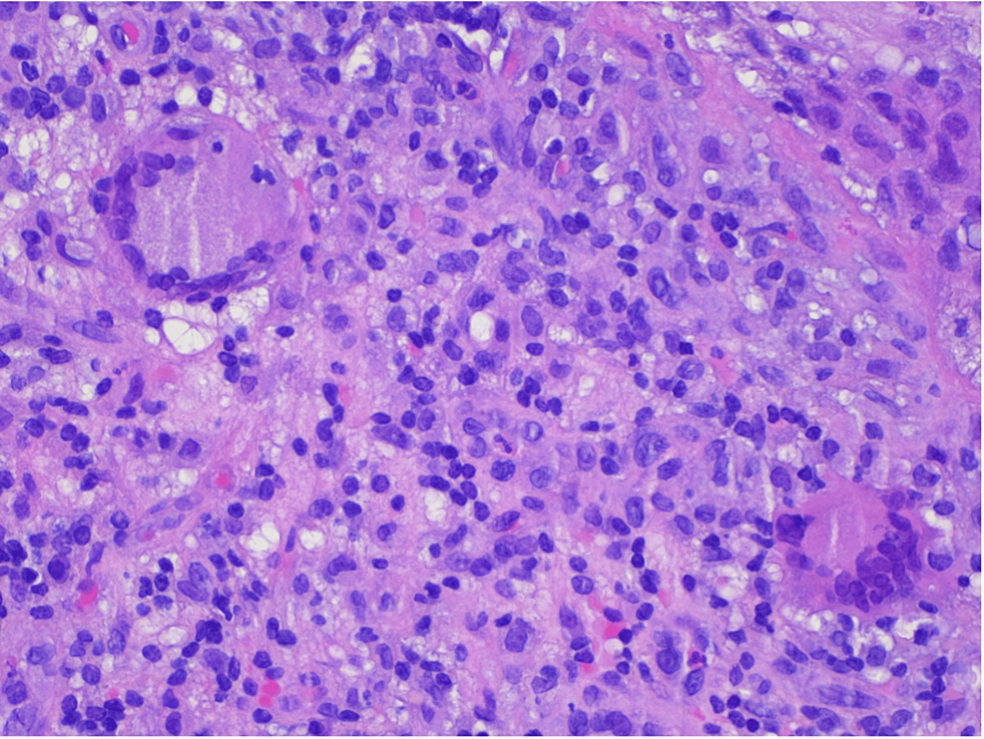
Anatomopathological evaluation of penobulbar urethra fragments H&E 400x magnification: lymphohistiocytic inflammatory cell infiltrate in the lamina propria of the urethral mucosa with scattered multinucleated giant cells (arrows)

From the etiological study of FUO carried out, serological tests for hepatitis B virus (HBV), hepatitis C virus (HCV), human immunodeficiency virus (HIV), and syphilis were negative. Levels of angiotensin-converting enzyme (ACE), immunoglobulins, sedimentation rate, and immunologic screening were normal. Serum and urinary flow cytometry showed no evidence of monoclonal peaks. The echocardiogram excluded endocarditis. The urine chemical and microscopic analyses showed no alterations. No acid-alcohol-fast-bacilli (AAFB) were observed in the patient's urine with the Ziehl-Neelsen stain. Polymerase chain reaction (PCR) screening for Mycobacterium tuberculosis deoxyribonucleic acid (DNA), along with urine culture tests for Mycobacteria, were negative. Bronchoscopy with bronchoalveolar lavage was performed, where Ziehl-Neelsen staining, mycobacterial culture, and Mycobacterium tuberculosis DNA screening by PCR in bronchial aspirate and bronchoalveolar lavage (BAL) samples were also negative. Cytological analysis of BAL and bronchial aspirate identified multinucleated giant cells without malignant cells (Figures 2, 3). Bronchoalveolar lavage demonstrated lymphocytic (31%) and neutrophilic (54%) alveolitis, with a CD4/CD8 ratio exceeding 3.5. Additionally, anatomopathological evaluation of penobulbar urethra fragments revealed squamous mucosa with ulceration, mixed inflammatory infiltrate, and multinucleated giant cells. The Ziehl-Neelsen stain was negative. No cellular atypia or signs of malignancy were identified (Figures 4, 5). 

To rule out the potential impact of sarcoidosis on other organs, an electrocardiogram, echocardiogram, cranioencephalic CT scan, and PET scan were conducted, all showing no abnormalities. The ophthalmologic examination revealed no pathological findings. Additionally, levels of thyroid-stimulating hormone, thyroxine, parathyroid hormone, and vitamin D were within the normal range. 

In light of these circumstances, a confirmed diagnosis of sarcoidosis with both pulmonary and extrapulmonary manifestations was established. The patient, previously treated for pulmonary tuberculosis, exhibited a positive result in the interferon-gamma release assay (IGRA). Treatment for latent tuberculosis was initiated, involving a daily regimen of 300 mg of isoniazid and 600 mg of rifampicin for three months, coupled with the initiation of corticosteroids at a daily dosage of 20 mg for sarcoidosis. Following discharge, the patient underwent follow-up at an outpatient internal medicine clinic, with a reevaluation scheduled one month later, showing sustained asymptomatic status. Subsequent monthly reassessments were planned to gradually taper corticosteroid therapy after two months of disease stability.

## Discussion

Certain forms of apparently non-infectious granulomatous inflammation may underlie the manifestation of fever of unknown origin (FUO). Clinical manifestations might resemble recognizable patterns of illness, such as sarcoidosis. However, characteristic findings of these disorders are often absent, even when non-caseating granulomas are present in various tissues. Individuals experiencing this type of idiopathic granulomatosis typically present symptoms like fever, sweats, malaise, arthralgias, myalgias, and weight loss. Although our patient did not exhibit elevated liver enzyme levels, these are usually heightened in approximately 60% of cases [8,10].

Diagnosing this disorder requires a biopsy of the affected tissue and the exclusion of other conditions showing this histologic pattern, such as tuberculosis, specific disseminated fungal infections, and lymphoma. In most cases (84%), the condition is restricted to thoracic findings, with only a minimal proportion (0.2%) of clinically diagnosed cases and 5% of postmortem diagnoses involving the urogenital system [11].

After reviewing the literature, two clinical cases have been documented of urethral involvement in sarcoidosis. In the first case, a 60-year-old African American female presented with progressive, irritative, and obstructive voiding symptoms. The diagnosis was confirmed through a transurethral biopsy. In the second case, sarcoidosis was discovered incidentally during endometrial curettage for an unrelated issue. Initially mistaken for a urethral carcinoma due to its appearance as a mass in the urethra, the biopsy revealed it to be an inflammatory mass caused by sarcoidosis. In both cases, systemic steroids were utilized as the primary treatment, leading to clinical improvement [6].

The case presented here is uncommon, being one of the few instances reported in the literature of urethral sarcoidosis as the initial manifestation. As stated, the patient's clinical manifestations had already been initiated by at least 2016, with the diagnosis only confirmed in 2023, highlighting the complexity involved in reaching a diagnosis. In our case, ACE levels were within the normal range, even though they are typically elevated in 75% of untreated individuals with active sarcoidosis. Although serum markers have a limited role in diagnosis, the low sensitivity and specificity of elevated ACE levels pose challenges, leading to false-positive rates of around 15% [5]. Bronchoalveolar lavage, when showing lymphocytosis of at least 15% and a CD4:CD8 T-lymphocyte ratio greater than 3.5, can provide supportive evidence for a sarcoidosis diagnosis [12].

Nevertheless, since there are no definitive diagnostic tests specific to sarcoidosis, establishing a diagnosis depends on excluding other potential causes [5]. Histopathological confirmation plays a vital role in achieving a conclusive diagnosis. Typically, if the symptoms and signs are consistent, the presence of non-caseating granulomas in at least one organ is satisfactory for the diagnosis, under the assumption of involvement in other organs. In our patient's case, the combination of imaging findings (hilar adenopathy), alterations in bronchoalveolar lavage, and the presence of multinucleated giant cells in both the lavage cytology and urethral fragments were crucial for the diagnosis. This allowed for the initiation of targeted therapy, leading to substantial clinical improvement. 

## Conclusions

Fever of unknown origin may arise from various causes, and it is essential to contemplate the possibility of sarcoidosis as a diagnosis when more common factors have been eliminated in clinical deliberation, emphasizing the critical need for a thorough diagnostic approach. While the identification of non-caseating granulomas forms the cornerstone for diagnosing sarcoidosis, the absence of pathognomonic features necessitates comprehensive investigations. 

While genitourinary sarcoidosis is relatively rare, it can manifest in any organ within the genitourinary tract, presenting varied symptoms that may resemble other diseases. This emphasizes the importance of including it in the differential diagnosis of urological conditions. Therapeutically, corticosteroids play a central role, with surgical interventions reserved for cases involving obstructive uropathy or when considering an alternative diagnosis of malignant disease. Recognizing these possibilities has the potential to prevent unnecessary hospitalizations and costly diagnostic tests and treatments. 
